# Dr. C. A. Harris

**Published:** 1860-10

**Authors:** 


### EDITORIAL DEPARTMENT.

Dr. C. A. Harris, the founder of this Journal and of the Baltimore Dental College, has gone to his rest—that rest prepared of old for such as he was. We find it next to impossible to speak of our loss. It is too recent, too overpowering, and of too direct personal concern to us for expression in words. Still, we cannot pass it by without some notice, however feeble and imperfect.

If we were to attempt to describe the character of our late most estimable friend, we should be able to comprise it in one word—"goodness." Amiability was his predominant trait, or something better than amiability, that christian charity which "thinketh no evil," which "suffereth long and is kind," which "is not easily provoked." It was impossible for any man to make an enemy of Dr. Harris. His generous heart forgave before a pardon was asked; plead against his judgment in behalf of those who had injured him, and was ingenious in finding excuses for all transgressions against him. He was one of those rare beings who "wrote his wrongs in water," who not only never cherished, but seemed incapable of feeling resentment. To his friends he was completely devoted; he never forgot them nor omitted any opportunity of rendering them a service. He was never satisfied unless he was expending his time, his money, or his influence in behalf of some one else. His whole life was one long act of benevolence. Pure as an altar-flame it rose, and has now lost itself in that heaven towards which it ever ascended.

It would speak ill, indeed, for human nature, were such a man to pass away without leaving many mourners. His loss is sincerely lamented by all who knew him, especially by those more immediately associated with him. We subjoin some of the testimonials which have reached us.

The Faculty of the Baltimore College of Dental Surgery, at a special meeting, convened on the 17th of October, passed the following resolutions:

*Resolved*, That the Faculty have received, with sincere sorrow, the intelligence of the decease of Prof. C. A. Harris, late President of this College.

*Resolved*, That as the projector of Collegiate Dental Education, and the laborious and successful founder of this College, Dr. Harris deserved the highest regards of the profession and the gratitude of the public. It may be said of him that he united Dental Surgery to Science, and raised the practice of it to the worth and respectability of a profession.

*Resolved*, That by the death of Prof. Harris this Faculty have lost a most valuable member—a man wise in counsel, pure in purpose and able in action.

*Resolved,* That by the death of Dr. Harris the several members of this Faculty have lost a highly esteemed friend—one of the most amiable and kind of human beings, warm and tenacious in friendship, almost incapable of anger, wholly incapable of resentment—an embodiment of the "charity that thinketh no evil," and of the humility that "esteemed others better than himself"—a man noble by nature and refined by grace—a gentleman by creation and a Christian without guile.

*Resolved,* That the Faculty will wear the usual badge of mourning during the College session.

*Resolved,* That a copy of these resolutions be sent to the family of Dr. Harris as an expression of sympathy in their great sorrow.

P. H. AUSTEN, *Dean of the Faculty.*

---

The Dentists of New York and its vicinity held a meeting, the proceedings of which, in accordance with the request contained in the 8th Resolution, we copy.

Pursuant to call, a meeting of the dentists of New York and vicinity took place at the rooms of Messrs. Jones & White (which were kindly offered for the occasion,) on Monday evening, Oct. 8th, at 8 o'clock.

On motion of Dr. John Allen, Dr. Eleazar Parmly was called to the chair, and Dr. Solyman Brown appointed Secretary; when the Chairman proceeded to make the following remarks in reference to the sad occasion that had called them together:

"*Gentlemen of the Dental Profession:*—I am glad to see so large a number brought together on this occasion, and, permit me, first, to thank you for the honor you have done me, and for your friendly consideration in selecting me to preside at this meeting. There is, perhaps, no one present who has had the same opportunity of knowing the deceased that I have had. My long acquaintance with Dr. Harris, in the societies to which we belonged, and my more intimate connection with him in the Baltimore College of Dental Surgery, while I held the office of Provost in that institution, enable me to say, with truth, that I have never known a man of more generous impulses, or more genial feelings, than he whose death has awakened our sympathies, and brought so many of his professional brethren together to express their high respect for his professional character, their admiration of his attainments, and their exalted esteem for his moral, social, and personal worth.

As one to whom he was long known and endeared by the many virtues that adorned his character, I trust it will not be deemed unbecoming in me to give this testimony concerning one who has labored more arduously as a practitioner, more untiringly as a writer, and more devotedly as a teacher of the Principles and Practice of Dental Surgery, than any person who has in any way, or in any country, ever been connected with our professional art.

In his domestic relations, Dr. Harris was exceedingly happy. His home was one of the most hospitable and delightful that I have ever known.

His house and his heart were always open to all those who approached him with the remotest claim upon his benevolence and bounty, with a widely extended philanthropy.

On motion, the Chair appointed a Committee of three to draft a series of resolutions expressive of the feelings of this meeting on the present occasion. Drs. Ambler, Brown and Foster were appointed.

While the Committee were absent, the Chair suggested that the gentlemen present should all sign the original call for the meeting, and remarked that there were already more names appended to it than there were dentists in the United States when he first commenced practice, in 1815.

The Committee returned and reported a Preamble and Resolutions. On motion, the report was accepted by the meeting, and the Committee dismissed.

On motion, the meeting proceeded to take up the resolutions *seriatim*, and Nos. 1, 2, 3, and 4 were adopted without dissent. Considerable debate followed the reading of the fifth resolution, and various suggestions and amendments were offered by different gentlemen present. The resolution amended by Dr. Rich, so as to change the number of the Committee from twenty to three, was finally adopted by the meeting. The other resolutions were then read and adopted, when on motion, the report as amended was adopted as a whole, as follows:

*Preamble*, Every distinct profession in human society has its leading members, men of energy, talent and eminence. This is as true of the Dental profession as of any other, and not less true in America than in other quarters of the globe.

The names of Greenwood, Wooffendale, Gardette, Hayden, Flagg, Hudson, Koecker, and Randall, among others that have left their sublunary labors, are evidences of this fact.

It has become our melancholy duty, in pursuance of the objects of this meeting, to add another name to this catalogue, more highly distinguished than any of his predecessors, for numerous and valuable contributions to the science and literature of his profession, as well by his writings as by personal inculcations as a teacher at the head of the oldest, and, for a long period, the only Dental College in the world.

Chapin A. Harris has gone to his rest. On Saturday, the 29th of the last month, he fell asleep, to awake in an endless Sabbath. His family, his profession, and his country are left to mourn his loss. We, therefore, offer to this meeting, composed of some of his professional brethren, the following:

*Resolved, First,* That, in the death of Dr. C. A. Harris,, removed from his earthly labors in the prime of manhood; from his family in the fervor of affection; and from his country in the ardor of his usefulness; we recognize a sad bereavement, which the members of the Dental profession in all civilized lands have just occasion to deplore.

*Resolved, Second,* That the labors of Dr. Harris, in assisting to establish the American Journal of Dental Science, the American Society of Dental Surgeons, and the Baltimore College of Dental Surgery, as well as in the construction and publication of his Dictionary of Medicine and Dentistry, and his Principles and Practice of Dental Surgery, have conferred favors on his profession which gratitude can never repay, because these and other labors in the same direction, have broken his health, abridged his life, and gathered his friends and his family around his early grave.

*Resolved, Third,* That a committee of three be appointed by this meeting to convey to the family of the departed, the sympathy of its members, on occasion of their great and irreparable loss.

*Resolved, Fourth,* That, in view of the great and important services which the self-denying labors of Dr. Harris, have rendered to the Dental profession, a suitable testimonial be presented to the family of the deceased, by such members of the profession as may choose to contribute to that object.

*Resolved, Fifth,* That a Committee of three be appointed by this meeting to invite all dentists to unite with us in subscribing to raise a money testimonial, to be presented to the widow of the late Dr. Harris, and that this Committee have power to appoint sub-Committees in Europe and America to carry out the above object.

*Resolved, Sixth,* That the said Committee be instructed to give ample notice of the purpose above explained, not only in all the Dental Journals, but also by means of a printed circular forwarded by mail to all the dentists in the United States, appointing the time for the presentation of said testimonial to the family, and determining the manner in which the fund shall be appropriated to its object.

*Resolved, Seventh,* That the Committee hereby authorized to receive and appropriate the contributions made for the benefit of the "Harris Testimonial Fund," shall be instructed to use no part of said fund for any expenses except for printing and mailing the circulars and letters sent to members of the profession, editors of periodicals, and members of the Harris family.

*Resolved, Eighth,* That this meeting hereby requests all editors of Dental periodicals to publish the proceedings of this meeting, together with the circular issued by the Committee, free of charge.

Solyman Brown,
John G. Ambler,
J. H. Foster,
} *Committee on Resolutions.*

On motion, the Committee of three mentioned in the fifth resolution, was appointed, consisting of Drs. Eleazar Parmly, Solyman Brown and E. J. Dunning. On motion, a Committee was appointed to transmit the resolutions adopted by this meeting to the family of the deceased, Dr. Harris. The officers of the meeting, assisted by Dr. Foster, were appointed a Committee. A resolution of thanks was then voted to the officers of the meeting, and briefly responded to by the Chair, after which the meeting adjourned.

During the proceedings, Drs. Parmly, Rich, Allen, Hill, Gunning, Roberts, Clarke, Castle, Franklin, Burrows, and others, took occasion to express their sympathies with the occasion which had called them together, and their deep appreciation of the worth of the late Dr. Harris and his labors. The meeting was well attended, some fifty of the most prominent members of the profession in New York being present."

A full and complete biography of the late Dr. C. A. Harris, is in course of preparation, and will appear in a future number.

